# Effectiveness of a blended booster programme for the long-term outcome of cognitive behavioural therapy for MS-related fatigue: A randomized controlled trial

**DOI:** 10.1177/13524585231213258

**Published:** 2023-11-29

**Authors:** Marieke de Gier, Heleen Beckerman, Jos WR Twisk, Hans Knoop, Vincent de Groot

**Affiliations:** Department of Medical Psychology, Amsterdam UMC, location Vrije Universiteit Amsterdam, Amsterdam, The Netherlands; Amsterdam Neuroscience Research Institute, Amsterdam, The Netherlands; Amsterdam Public Health Research Institute, Amsterdam, The Netherlands; Department of Rehabilitation Medicine, MS Center, Amsterdam UMC, location Vrije Universiteit Amsterdam, Amsterdam, The Netherlands; Amsterdam Public Health Research Institute, Amsterdam, The Netherlands; Department of Epidemiology and Data Science, Amsterdam UMC, location Vrije Universiteit Amsterdam, Amsterdam, The Netherlands; Department of Medical Psychology, Amsterdam UMC, location Vrije Universiteit Amsterdam and location University of Amsterdam, Amsterdam, The Netherlands; Amsterdam Public Health Research Institute, Amsterdam, The Netherlands; Department of Rehabilitation Medicine, MS Center, Amsterdam UMC, location Vrije Universiteit Amsterdam, Amsterdam, The Netherlands; Amsterdam Neuroscience Research Institute, Amsterdam, The Netherlands

**Keywords:** MS-related fatigue, cognitive behavioural therapy, e-health, booster sessions, long-term effects, randomized controlled trial

## Abstract

**Background:**

Cognitive behavioural therapy (CBT) reduces MS-related fatigue. However, studies on the long-term effects show inconsistent findings.

**Objective:**

To evaluate whether a blended booster programme improves the outcome of CBT for MS-related fatigue on fatigue severity at 1-year follow-up.

**Method:**

A multicentre randomized clinical trial in which 126 patients with MS were allocated to either a booster programme or no booster programme (control), after following 20-week tailored CBT for MS-related fatigue. Primary outcome was fatigue severity assessed with the Checklist Individual Strength fatigue subscale 1 year after start of treatment (T52). Mixed model analysis was performed by a statistician blinded for treatment-allocation to determine between-group differences in fatigue severity.

**Results:**

Fatigue severity at 1-year follow-up did not differ significantly between the booster (*N* = 62) and control condition (*N* = 64) (*B* = −2.01, 95% confidence interval (CI) = −4.76 to 0.75). No significant increase in fatigue severity was found at T52 compared with directly post-treatment (T20) in both conditions (*B* = 0.44, 95% CI = −0.97 to 1.85).

**Conclusion:**

Effects of CBT were sustained up to 1 year in both conditions. The booster programme did not significantly improve the long-term outcome of CBT for MS-related fatigue.

**Trial registration:**

Dutch Trial Register (NTR6966), registered 18 January 2018 https://www.trialregister.nl/trial/6782

## Introduction

Fatigue is a highly prevalent and burdensome symptom in multiple sclerosis (MS). MS-related fatigue can be effectively treated with cognitive behavioural therapy (CBT).^1,2^ Meta-analyses show that although CBT leads to a reduction of fatigue directly post-treatment with moderate to large effect sizes,^1,2^ long-term effects are less clear. In some randomized clinical trials (RCTs) treatment effects are partly maintained until 3–6 months after treatment^3,4^ or even improve further during the 4 months after treatment after which effects were maintained up till 1 year.^5,6^ However, in one study treatment effects gradually wore off during the 8 months post-treatment.^7^ These differences in long-term effects are not yet understood, but differences in treatment protocol, treatment format and duration of CBT, may play a role in the differences in outcomes between studies.

Little is known about the mechanisms involved in the relapse in fatigue over time after CBT. Mediation analysis showed that increased daytime sleepiness, more problems in staying physically active and an increase of subjective cognitive problems mediated relapse in fatigue following CBT in patients with MS.^8^ In order to maintain treatment effects, the changes in behaviours and cognitions attained during CBT need to be sustained after treatment. Especially in a progressive condition such as MS, where patients over time are confronted with new symptoms, preventing relapse in unhelpful cognitions and behaviour patterns may be challenging. Therefore, booster sessions may be helpful in sustaining behaviour change and improving long-term outcomes of CBT.

Although offering booster sessions has been suggested as a way to improve sustainment of treatment effects, few studies report on their effectiveness. Effectiveness of boosters varies between studies^9,10^ and comparison is complex and likely to be dependent on the behaviour or symptoms targeted in treatment, the type and duration of the initial intervention, duration and content of the booster intervention, among other factors. To date, effectiveness of booster sessions after CBT for fatigue has not been studied.

The aim of the present RCT was to study whether a booster programme improves the outcome of CBT for MS-related fatigue at 1-year follow-up.

## Method

### Trial design

**Figure 1.**
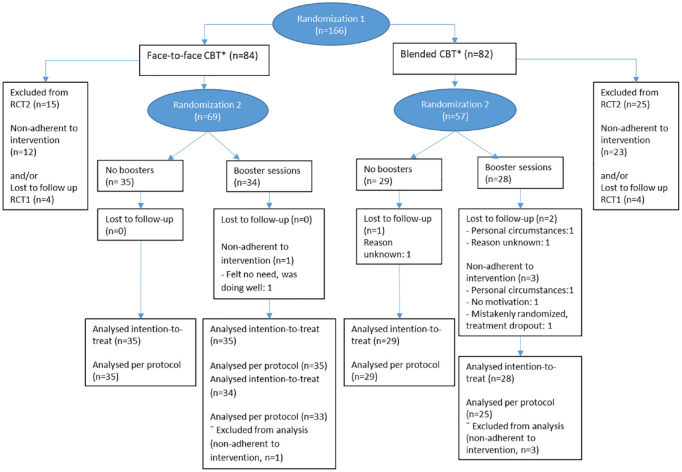
Consort flow diagram. *Non-inferiority RCT (RCT1).

This superiority RCT followed after a non-inferiority RCT, in which patients received either face-to-face or blended CBT. Blended CBT was non-inferior to face-to-face CBT with respect to its positive effect on fatigue severity.^11^ Participants were randomized again post-treatment (at 20 weeks) to either a condition with boosters or a condition without boosters (see Figure 1).^12^ Participants in the non-inferiority RCT met the following inclusion criteria: (a) definitive diagnosis of MS, (b) severely fatigued, checklist individual strength (CIS) fatigue ⩾35, (c) aged between 18 and 70 years, (d) ambulatory, Expanded Disability Status Scale (EDSS ⩽6), (e) no evident signs of an exacerbation and no corticosteroid treatment in the past 3 months and (f) no clinical indication of current infections, anaemia or thyroid dysfunction. The exclusion criteria were (a) depressive disorder, assessed with the Beck Depression Inventory-Primary Care version (BDI-PC)^13^ and Mini-International Neuropsychiatric Interview (M.I.N.I.),^14^ (b) primary sleep disorders, (c) other severe somatic or psychiatric co-morbidity (Cumulative Illness Rating Scale^15^ item ⩾ 3), (d) current pregnancy or having given birth in the past 3 months, (e) pharmacological treatment for fatigue that was started in the past 3 months, (f) non-pharmacological therapies for fatigue in the past 3 months and (g) having received CBT for fatigue.

Only participants who adhered to the initial treatment period of 20 weeks were included in the booster trial. Adherence was defined as (a) having started treatment and (b) having finished the initial treatment period with a face-to-face session and assessment at T20.^12^

### Randomization

Randomization with concealed treatment allocation was carried out by a project member not involved in the enrolment or assessments. A web-based randomization facility within CASTOR EDC was used, with a computer-generated randomization scheme and stratification for treatment centre and initial treatment condition in the first RCT, and using random variable block sizes (range 2–6). The outcome of the randomization was reported to the therapist by the research psychologist.

The study was approved by the Medical Ethical Review Committee, Amsterdam University Medical Centres, location Free University (registration no. 2017.538, NL62622.029.17), and by the local ethical committees of the participating hospitals and rehabilitation centres. The study was registered in the Dutch Trial Register (NTR6966). The study protocol has been published.^12^

### Intervention

A blended booster programme called ‘MS Stay Fit’ was developed, consisting of online booster modules and two booster video consultations with the same therapist as in the preceding CBT at 2 and 4 months after finishing the 20-week CBT for MS-related fatigue. The booster programme was aimed at preventing fatigue relapse, and eventually reaching treatment goals that were not fully met during the first 20 weeks of treatment. ‘MS Stay Fit’ consisted of five online ‘booster modules’ aimed at the factors that mediated the relapse in fatigue in the TREFAMS-CBT trial,^8^ that is, sleep–wake pattern and activity regulation but also at cognitions about fatigue and coping with ‘normal fatigue’. The content of the booster programme is described in detail in Supplement 1.

Patients in the control condition did not receive the booster programme, but ended treatment after the last treatment sessions at 20 weeks.

### Adherence

Therapists registered whether patients attended the booster consultations and whether they completed the homework assignments in MS Stay Fit. Log data of the online platform provided information about which booster modules were opened and completed. Treatment adherence for the booster condition was defined as attending at least one booster consultation, since some patients may not need a second booster session in case they did not profit from CBT, or if they felt confident about sustaining treatment effects. Reasons for not attending a second booster session were noted.

All study participants were requested to complete the follow-up questionnaires at weeks 39 and 52.

### Outcomes

All outcome measures were assessed online at baseline before randomization in the non-inferiority study (T0), at the end of the 20-week treatment period prior to randomization for the current RCT (T20), 9 months and 1 year after the start of treatment (T39 and T52), respectively.

Primary outcome was fatigue severity assessed with the CIS fatigue severity subscale. The CIS is a 20-item fatigue questionnaire, consisting of 4 subscales assessing fatigue severity (8 items), reduction in motivation, reduction in physical activity and concentration problems.^16^ The items are answered on a 7-point Likert-type scale, leading to a total score of the fatigue severity subscale between 8 and 56 points. A score of 35 or higher is indicative for severe fatigue.^17^ The CIS is a reliable and valid instrument.^16^ Clinically significant improvement was defined as either a score of <35 on the CIS subscale fatigue severity, or an improvement of ⩾8 points on this subscale.^12^

Secondary outcome measures were other fatigue measures (Fatigue Severity Scale and PROMIS-Fatigue Short Form 8a^18–20^), limitations in daily functioning and quality of life (Sickness Impact Profile, Work and Social Adjustment Scale and SF36^21–24^). Psychometric qualities of these questionnaires are described in Supplement 2.

### Sample size

In the TREFAMS-CBT study an immediate treatment effect of 6.7 points and a subsequent fatigue relapse of 6.7 points during follow-up was found.^7^ The boosters were aimed at preventing this relapse, leading to an expected between-group difference of 6.7 on the CIS fatigue subscale at 1-year follow-up. Two-sided significance testing with an α of 5%, two study groups of 75 participants at 1-year (needed for the initial non-inferiority RCT), would result in a power of 98%.

Sample size was, however, determined by the number of participants finishing the original treatment period meeting the inclusion criteria for the second part of the RCT (*N* = 126). Based on an SD of 11 on the CIS fatigue post-treatment, a power of 80% and an α of 5%, a difference of 5.5 on the CIS fatigue subscale would be statistically significant in two groups of 62 patients.

### Blinding

Patients and therapists could not be blinded for allocation. The research assistant responsible for the assessments and the statistician conducting the statistical analyses were blinded for initial treatment format and booster condition.

### Statistical analyses

#### Primary analysis

First, to determine whether there was an effect of boosters, the primary outcome was analysed using a linear mixed model analysis, including CIS-fatigue (at both T39 and T52) as the dependent variable, booster (yes/no) and the CIS-fatigue at T20 as (fixed) covariates. To determine the effect of boosters on fatigue severity compared with no boosters at T39 and T52 separately the linear mixed model analysis was extended with time and boosters by time interaction as (fixed) covariates.

All mixed model analyses were conducted with four levels: measurements were clustered within patients, patients were clustered within therapists and therapists were clustered within treatment centres.

The analysis was repeated with the initial treatment condition in the non-inferiority RCT (F2F or blended CBT), and the interactions between initial treatment condition and booster condition, to investigate whether effectiveness of booster sessions differ between face-to-face CBT and blended CBT.

In addition, to determine the course of fatigue severity post-treatment for the whole sample, a linear mixed model analysis was conducted, including CIS fatigue (at T20, T39 and T52) as the dependent variable, and time as (fixed) covariates. In case of a significant effect of boosters in the primary analysis, booster condition was added in the analysis.

#### Secondary analyses

The analysis was repeated for the secondary outcomes with the T20 measure of the outcome as covariate.

The proportion of clinically significantly improved patients assessed with the CIS fatigue subscale in both conditions was also compared with a logistic regression analysis with clinically significantly improved as dichotomous dependent variable, and booster condition and clinically relevant improved (T20) as fixed covariates. Clinically significant improvement was defined as either a score of less than 35 on the CIS20r fatigue severity subscale or an improvement of at least 8 points on this subscale compared with baseline (T0).

Analyses were based on the intention-to-treat sample. In case of a large attrition rate (>10%) the analyses were to be repeated on the per protocol sample as well, including participants adherent to the booster allocation.

## Results

### Patients

Recruitment and inclusion of participants took place between April 2018 and December 2021.^11^ Of the 166 participants in the non-inferiority RCT, 126 participants (77% female) with a mean age of 44.9 and a median disease duration of 7.0 years at baseline were subsequently randomized in the present study to either booster sessions or no booster programme. Reasons for exclusion were non-adherence to the initial treatment or lost to follow-up in the non-inferiority RCT and are displayed in the flow diagram (Figure 1). Patient characteristics are displayed in Table 1. Three patients were lost to follow-up at T52: one in the control condition and two in the booster condition. In the booster condition adherence was 94%. The reasons for non-adherence are described in the flow diagram (Figure 1), and included no motivation or need for boosters (*N* = 2) or personal circumstances (*N* = 1). Fifty patients attended both booster sessions, while eight patients attended only one. One adherent patient did not make use of MS Stay Fit, although attending both online booster sessions with the therapist.

**Table 1.** Characteristics of the 126 participants with MS-related fatigue in the booster RCT.

	No boosters (*n* = 64)	Boosters (*N* = 62)
Age (year) (mean, SD)	44.0 (10.9)	45.9 (10.6)
Gender		
Female	47 (73.4%)	50 (80.6%)
Male	17 (26.6%)	12 (19.4%)
Level of education		
Low	6 (9.4%)	3 (4.8%)
Medium	27 (42.2%)	25 (40.3%)
High	31 (48.4%)	34 (54.8%)
Civil status		
Living alone	11 (17.2%)	8 (12.9%)
Living with partner	46 (71.9%)	49 (79.0%)
Time since diagnosis (year) (median, min–max)	7.0 (0 to 41)	7.0 (0 to 29)
EDSS (median, min–max)	3.5 (0 to 6)	3.5 (1 to 6)
Type of MS		
RRMS	50 (78.1%)	50 (80.6%)
PPMS	6 (9.4%)	6 (9.7%)
SPMS	6 (9.4%)	6 (9.7%)
Unknown	2 (3.1%)	0
Scores after initial treatment (at T20)		
CIS fatigue (T20)	30.9 (10.8)	33.3 (11.5)
SIP8 total (T20)	1086.9 (641.4)	1136.5 (798.2)
SF36 physical functioning (T20)	72.7 (19.0)	67.8 (24.7)
WSAS (T20)	15.5 (6.4)	16.0 (8.3)

MS: multiple sclerosis; RCT: randomized clinical trial; EDSS: Expanded Disability Status Scale; RRMS: relapsing remitting multiple sclerosis; PPMS: primary progressive MS; SPMS: secondary progressive MS; CIS: Checklist Individual Strength; SIP: Sickness Impact Profile; SF36: short form 36; WSAS: Work and Social Adjustment Scale.

The booster sessions were provided by the same therapist as the initial treatment. In total, 25 therapists from 14 participating rehabilitation centres and hospitals treated patients in the booster RCT.

Considering the small attrition rate, no additional per protocol analyses were conducted.

### Effect of booster sessions on fatigue severity and secondary outcomes after 1 year

There was no significant difference in fatigue severity between the booster and control condition at T39 and T52 (overall *B* (average over both time points) = −1.42, 95% CI = −3.81 to 0.98; T39 *B* = −0.81, 95% CI = −3.58 to 1.96; T52 *B* = −2.01, 95% CI = −4.76 to 0.75). This was independent of the initial treatment condition (face-to-face vs. blended CBT) as there was no significant interaction effect with initial treatment condition on the effect of the boosters on fatigue (*p* = 0.551). All outcome measures on T20, T39 and T52 and differences between booster and control groups at T39 and T52 are reported in Table 2.

**Table 2.** Scores on primary and secondary outcomes at all time points and the between-group effects.

	No boosters			Boosters			Between-group effect at T39 corrected for T20	Between-group effect at T52 corrected for T20
	T20 (*n* = 64)	T39 (*n* = 62)	T52 (*n* = 63)	T20 (*n* = 62)	T39 (*n* = 59)	T52 (*n* = 60)		
	Mean (SD)	Mean (SD)	Mean (SD)	Mean (SD)	Mean (SD)	Mean (SD)	*B* (95% CI)	*B* (95% CI)
CIS fatigue	30.9 (10.8)	32.5 (11.8)	32.6 (10.7)	33.3 (11.5)	33.4 (13.1)	32.3 (11.9)	−0.81 (−3.58 to 1.96)	−2.01 (−4.76 to 0.75)
FSS	4.0 (1.1)	4.1 (1.0)	4.0 (1.1)	4.4 (1.2)	4.2 (1.4)	4.1 (1.4)	−0.22 (−0.57 to 0.13)	−0.13 (−0.48 to 0.22)
PROMIS-fatigue	20.4 (6.0)	21.8 (6.9)	21.1 (6.8)	22.7 (7.1)	21.8 (7.9)	22.0 (7.7)	−1.68 (−3.53 to 0.17)	−0.93 (−2.77 to 0.91)
SF36								
Physical funct.	72.7 (19.0)	71.4 (21.3)	72.1 (20.7)	67.8 (24.7)	69.8 (25.6)	68.6 (25.5)	2.92 (−1.91 to 7.75)	0.82 (−3.40 to 5.64)
Role physical	57.0 (40.5)	51.2 (41.4)	50.0 (37.9)	54.8 (38.6)	50.0 (41.7)	50.0 (40.3)	0.58 (−11.84 to 13.00)	2.36 (−10.01 to 14.73)
Role emotional	83.9 (32.0)	73.2 (38.9)	77.0 (35.8)	87.1 (27.2)	87.1 (27.3)	82.2 (34.3)	12.23 (0.45 to 24.02)	4.09 (−7.64 to 15.82)
Energy	59.1 (15.9)	57.1 (17.3)	56.2 (18.4)	54.0 (18.3)	54.6 (20.4)	54.9 (19.4)	1.73 (−3.23 to 6.70)	2.90 (−2.05 to 7.84)
Emo.well-being	78.6 (15.0)	77.8 (16.6)	79.1 (15.1)	76.6 (15.2)	74.9 (16.9)	75.4 (17.8)	−1.99 (−6.41 to 2.43)	−2.51 (−6.91 to 1.90)
Social funct.	76.2 (19.3)	73.0 (23.2)	75.8 (21.3)	68.5 (23.8)	67.5 (24.3)	68.5 (22.7)	−0.77 (−7.42 to 5.88)	−2.38 (−9.01 to 4.25)
Pain	74.8 (20.3)	75.4 (23.2)	73.2 (22.9)	72.4 (19.3)	75.8 (22.9)	74.4 (20.8)	2.23 (−3.94 to 8.39)	3.22 (−2.93 to 9.36)
General health	52.8 (18.9)	55.9 (20.1)	53.3 (18.0)	49.9 (21.4)	50.5 (21.0)	50.3 (19.0)	−2.44 (−7.00 to 2.12)	−0.06 (−4.60 to 4.49)
SIP total	1086.9 (641.4)	1087.2 (668.1)	1035.1 (616.2)	1136.5 (798.2)	1167.2 (804.8)	1138.9 (829.9)	18.07 (−142.77 to 178.91)	70.19 (−97.67 to 238.06)
WSAS total	15.5 (6.4)	15.5 (8.2)	17.1 (8.9)	16.0 (8.3)	16.3 (8.5)	16.8 (9.1)	0.38 (−1.79 to 2.55)	−0.72 (−2.89 to 1.44)
BDI total	1.83 (2.1)	1.6 (1.8)	1.6 (1.9)	1.7 (1.8)	2.0 (2.4)	2.0 (2.8)	0.54 (−0.11 to 1.20)	0.49 (−0.16 to 1.14)
							Odds ratio (95% CI)	Odds ratio (95% CI)
cl.r.change^a^ (*n*)	49 (76.6%)	45 (72.6%)	45 (70.3%)	43 (69.4%)	37 (62.7%)	46 (74.2%)	0.71 (0.27 to 1.82)	2.08 (0.75 to 5.76)

CI: confidence interval; CIS: checklist individual strength; FSS: Fatigue Severity Scale; SF36: short form 36; SIP: Sickness Impact Profile; WSAS: Work and Social Adjustment Scale; BDI: Beck Depression Inventory.

a Clinically relevant change = either CIS fatigue post treatment < 35, or a minimal change of 8 points on the CIS fatigue.

No between-group differences were found on other fatigue measurements, daily functioning and quality of life after 1-year follow-up. The proportion clinically relevant change at T52 was 70.3% in the control condition (*n* = 64) and 74.2% (*n* = 62) in the booster condition, with no significant between-group differences in these numbers.

### Course of fatigue severity post-treatment

**Figure 2.**
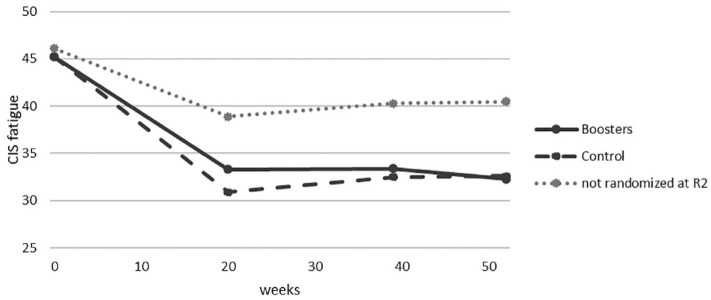
Fatigue severity from baseline to 1-year follow-up in the booster condition and control condition (no booster), and in the group of participants that were excluded from the second trial.

No significant change in fatigue severity was found on T39 (*B* = 0.87, 95% CI = −0.56 to 2.29) or T52 (*B* = 0.44, 95% CI = −0.97 to 1.85) compared with directly post-treatment (T20), indicating that no significant relapse in fatigue severity occurred. Figure 2 shows the course in fatigue severity from baseline until T52 in the booster- and control condition, and in the participants of the non-inferiority RCT, who were not randomized in the present study (*N* = 40). Congruence of treatment preference with the allocated initial treatment (face-to-face or blended CBT) did moderate treatment effect in the non-inferiority RCT.^11^ We found no significant effect of treatment preference on boosters (data not shown).

## Discussion

The aim of this study was to determine whether a blended booster programme could prevent fatigue relapse and improve long term effects of CBT for MS-related fatigue. No significant effect of the booster programme was found on fatigue severity and secondary outcomes at 1-year follow-up. Since fatigue severity did not increase in both conditions during the follow-up period, this study also indicates that wearing off of the positive effect of CBT on fatigue did not occur. No differences between booster- and control condition were found on secondary outcomes as well.

The absence of the expected effect of the booster programme, compared with the no booster control condition, is most likely explained by the fact that the control group did not show a relapse in fatigue severity. This latter finding was surprising, considering the relapse after successful treatment found in the TREFAMS-CBT study, using the same treatment protocol and inclusion criteria.^7^ The difference with respect to the long-term effects between the previous and current study is substantial. One possible explanation for this could be the difference in treatment length. The CBT in the present study had a treatment duration of 20-weeks compared with 16 weeks in the TREFAMS-CBT study.^7^ Spreading the same 12 therapy sessions over a longer treatment period provided more time for patients to attain their treatment goals and perhaps better sustain changes in fatigue-related cognitions and behaviour. Although similar treatment effects occurred within the 16-week treatment period, experiencing ‘normal’ fatigue levels for a longer period may further strengthen these changes and improve self-efficacy regarding fatigue.

However, when comparing the results with other RCTs on CBT for MS-related fatigue, there is no indication that treatment duration predicts long-term effects. Most studies had a treatment duration between 8 and 12 weeks, and also reported sustained treatment effects, although not all studies assessed patients up till 1 year.^3,4^ Considering the differences in treatment protocol, duration, treatment format and study designs, it remains difficult to distinguish the factors affecting the long-term outcome of CBT.

In the present study, therapists and patients knew that CBT was an effective treatment, but that relapses might occur. Mediation analysis had shown which factors mediated the effect of CBT and which factors mediated relapse in fatigue severity after 1 year.^8^ The aim of this study was specifically to improve long-term effects, with a stronger emphasis on these mediating factors during treatment. Furthermore, the proactive awareness of therapists may have played a role as well in the current study. All therapists were aware of the subsequent booster or control allocation during the last treatment session, which may have affected the emphasis on preventing fatigue relapse in the last treatment session. Therapists may have applied the knowledge about relapse prevention from the booster programme in the control condition as well.

At the time of the TREFAMS-CBT study (2010–2017), CBT for MS-related fatigue was not implemented in the Netherlands yet, and only a few international studies had shown positive outcomes of CBT for fatigue in MS. Fatigue was considered as a chronic symptom which was difficult to influence and treatment as usual was mostly aimed at coping with fatigue instead of reducing fatigue. During the present study, CBT was more solidly evidence-based, which may have affected the attitude and expectancies of therapists and patients in a positive way.

Maintaining treatment effects of fatigue interventions in chronic progressive diseases may be especially challenging, considering the fact that patients will be faced with ‘everyday’ fatigue, which in the context of the chronic disease may be perceived as a disease-specific symptom. This may increase the risk of dysfunctional coping strategies in response to fatigue and thereby increase the risk of perpetuating fatigue. Although the present study showed promising results in sustaining treatment effects, the follow-up period of 1 year after start of treatment is relatively short. No studies have reported treatment effects of CBT on fatigue severity in MS patients on a longer term than 1 year. So the longer-term course of fatigue and the effects of boosters are not known. It would be of clinical relevance to study the outcome of CBT over a longer follow-up period, and to get a better understanding of factors mediating the long-term effects of CBT for fatigue. Booster sessions could potentially be beneficial for patients at high risk for relapse in fatigue severity or dysfunctional behaviour patterns.

### Strengths and limitations

This is the first study testing the effectiveness of a booster programme in improving the long-term effects after CBT for MS-related fatigue. There are several limitations to take into consideration. Only participants who adhered to the initial treatment were included in the booster trial, which may have biased the long-term effects of CBT for fatigue in a positive way. Participants non-adherent to the treatment and excluded from present RCT showed higher fatigue levels at T20, which remained high at long-term follow-up. However, the course of fatigue severity of this group of participants shows no larger fatigue relapse compared with the participants who were randomized in the present RCT (Figure 2).

### Conclusion and clinical implications

Treatment effects of both blended and face-to-face CBT for MS-related fatigue were maintained up to 1 year after start of treatment. The booster programme did not improve long-term effects. Based on these findings overall clinical implementation of a booster programme is not indicated.

## Supplemental Material

sj-docx-1-msj-10.1177_13524585231213258 – Supplemental material for Effectiveness of a blended booster programme for the long-term outcome of cognitive behavioural therapy for MS-related fatigue: A randomized controlled trial Click here for additional data file. Supplemental material, sj-docx-1-msj-10.1177_13524585231213258 for Effectiveness of a blended booster programme for the long-term outcome of cognitive behavioural therapy for MS-related fatigue: A randomized controlled trial by Marieke de Gier, Heleen Beckerman, Jos WR Twisk, Hans Knoop and Vincent de Groot in Multiple Sclerosis Journal

sj-docx-2-msj-10.1177_13524585231213258 – Supplemental material for Effectiveness of a blended booster programme for the long-term outcome of cognitive behavioural therapy for MS-related fatigue: A randomized controlled trial Click here for additional data file. Supplemental material, sj-docx-2-msj-10.1177_13524585231213258 for Effectiveness of a blended booster programme for the long-term outcome of cognitive behavioural therapy for MS-related fatigue: A randomized controlled trial by Marieke de Gier, Heleen Beckerman, Jos WR Twisk, Hans Knoop and Vincent de Groot in Multiple Sclerosis Journal
