# Supplementary material for: Effectiveness of a blended booster programme for the long-term outcome of cognitive behavioural therapy for MS-related fatigue: A randomized controlled trial
Source: Mult Scler. 2023 Nov 29;30(1):71–9. doi: 10.1177/13524585231213258 (PMC10782645; doi:10.1177/13524585231213258)
Supplement: sj-docx-1-msj-10.1177_13524585231213258 – Supplemental material for Effectiveness of a blended booster programme for the long-term outcome of cognitive behavioural therapy for MS-related fatigue: A randomized controlled trial [file sj-docx-1-msj-10.1177_13524585231213258.docx]

**Supplement 1. TiDier Blended booster programme for CBT for MS-related fatigue**

**Blended booster programme “MS Stay Fit”**

**Why** Severe MS-related fatigue can be effectively treated with CBT. However, long term effects are unclear, and in the previous TREFAMS-CBT study testing the effectiveness of CBT for MS-related fatigue, treatment effects wore off within one year (1). For that reason, we developed a booster programme aimed at sustaining treatment effects and preventing relapse (2).

**What** An online booster programme, called MS Stay Fit, was developed, consisting of an introduction module and four optional booster modules, in which they can refresh what they learned during the CBT. These interactive modules were aimed at the factors that mediated the relapse in fatigue following CBT in the previous TREFAMS-CBT trial (3).

1. *Introduction* in which patients make a personalized plan aimed at sustaining the gains they have reached during treatment. This module also includes *Evaluation 1* and *Evaluation 2* which are schedules prior to the video consultations with the therapist, two and four months after the end of the initial treatment period. In these Evaluations patients can reset their plans and goals for the following months. In both the introduction and the evaluation sessions, coping with “normal fatigue” is addressed.
2. *Sleep-wake pattern*
3. *Activity regulation* (both physical and mental)
4. *Helpful thinking,* including focusing on fatigue
5. *Reaching goals*

MS Stay Fit was provided on a platform of “Minddistrict”.

At 2 and 4 months after the end of treatment, patients received a video consultation with their therapist, evaluating progress and eventual problems patients were facing in maintaining treatment effects.

**Who provided the therapy** Qualified psychologists were trained in applying the blended booster programme, during their 4-day training in the treatment manual of CBT for MS-related fatigue and applying blended CBT (MS Fit and MS Stay Fit) on the online platform of Minddistrict.

All CBT therapists completed a 4-day course on how to provide CBT in accordance with the TREFAMS-CBT protocol. Furthermore, the CBT therapists were supervised every second week by a clinical psychologist with experience in delivering CBT for MS-fatigue. All therapists (24 female and 2 male in 14 study centers) were equally qualified and trained in applying the blended booster programme. The number of therapists per treatment centre varied from 1 to 6 (4).

**How, when and how much** Patients received two 45-minute video consultations at 2 and 4 weeks after the end of the initial 20-week CBT period. In addition, they had access to the booster modules MS Stay Fit, until the last booster consultation at 4 months.

**Where** Patients could have the video consultations from home or where ever they pleased.

**Tailoring** The booster modules of MS Stay Fit were not patient tailored. However, the modules were optional, so patients could access them based on their preference or need.

**How well**: Therapists registered if patients attended the booster consultations and if they completed the homework assignments in MS Stay Fit. Log data of the online platform provided information about which booster modules were opened and completed by the patient. Treatment adherence for the booster condition was defined as attending at least one booster consultation.


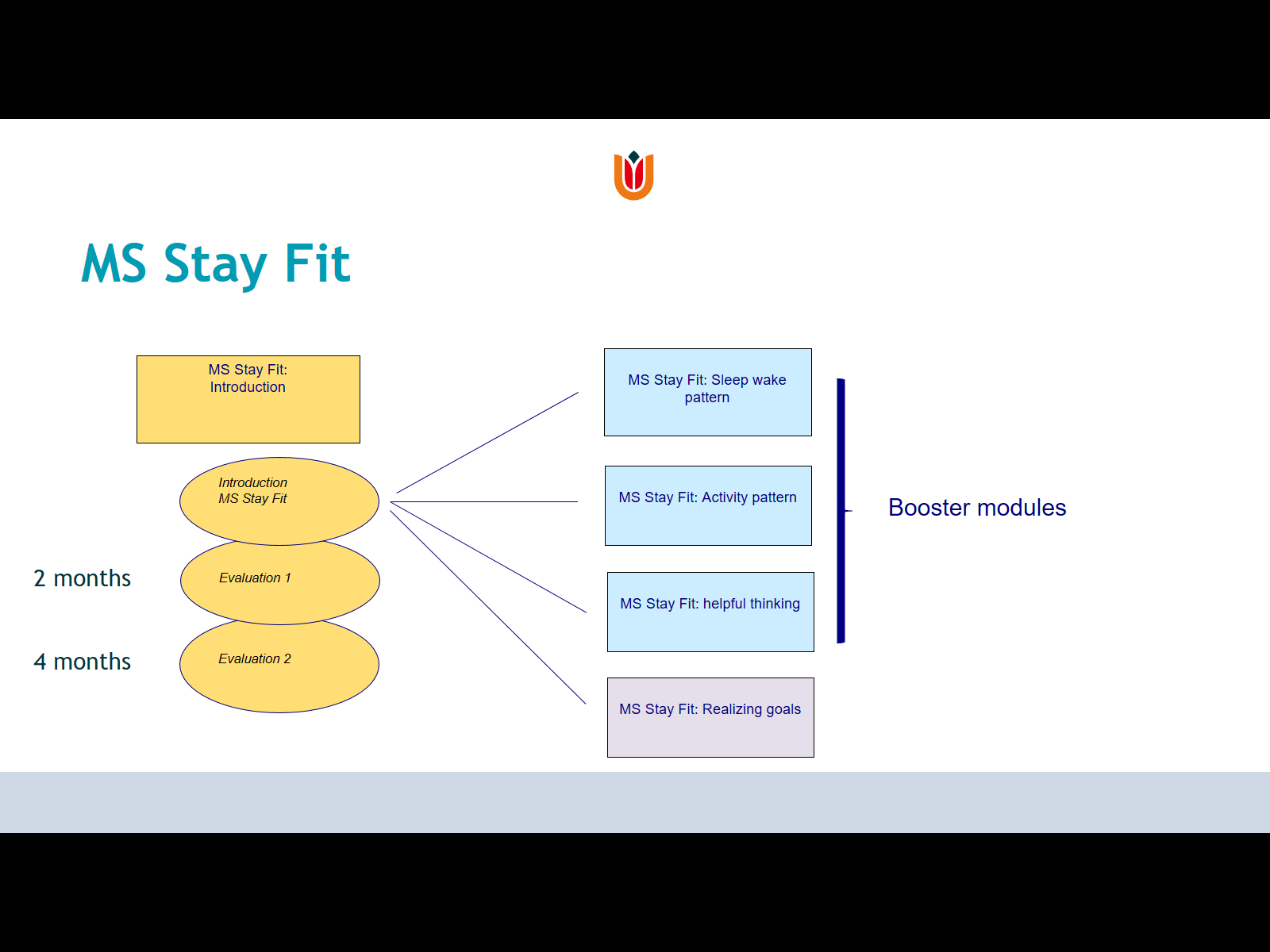


*Figure 1 MS Stay Fit*

**Control condition (no boosters)**

**Why** In order to study the effectiveness of booster sessions, the control group received no booster programme, meaning that the initial CBT ended after 20 weeks. Allocated participants had no further contact with their therapist until T52.

**How well**: For the control condition, adherence was defined as not receiving any additional treatment regarding fatigue after the initial 20-week CBT period.

**References**

1. van den Akker LE, Beckerman H, Collette EH, Twisk JW, Bleijenberg G, Dekker J, et al. Knoop H, de Groot V, Trefams-AcE study group. Cognitive behavioral therapy positively affects fatigue in patients with multiple sclerosis: Results of a randomized controlled trial. Mult Scler. 2017;23(11):1542-53.

2. Houniet-de Gier M, Beckerman H, van Vliet K, Knoop H, de Groot V. Testing non-inferiority of blended versus face-to-face cognitive behavioural therapy for severe fatigue in patients with multiple sclerosis and the effectiveness of blended booster sessions aimed at improving long-term outcome following both therapies: study protocol for two observer-blinded randomized clinical trials. Trials. 2020;21(1):98.

3. van den Akker LE, Beckerman H, Collette EH, Knoop H, Bleijenberg G, Twisk JW, Dekker J, de Groot V, Trefams-Ace study group. Cognitive behavioural therapy for MS-related fatigue explained: A longitudinal mediation analysis. J Psychosom Res. 2018;106:13-24.

4. de Gier M, Beckerman H, Twisk J, Knoop H, de Groot V. Blended versus face-to-face cognitive behavioural therapy for severe fatigue in patients with multiple sclerosis: A non-inferiority RCT. Multiple Sclerosis Journal. 2023:13524585231185462.
