# Supplementary material for: Effectiveness of a blended booster programme for the long-term outcome of cognitive behavioural therapy for MS-related fatigue: A randomized controlled trial
Source: Mult Scler. 2023 Nov 29;30(1):71–9. doi: 10.1177/13524585231213258 (PMC10782645; doi:10.1177/13524585231213258)
Supplement: sj-docx-2-msj-10.1177_13524585231213258 – Supplemental material for Effectiveness of a blended booster programme for the long-term outcome of cognitive behavioural therapy for MS-related fatigue: A randomized controlled trial [file sj-docx-2-msj-10.1177_13524585231213258.docx]

**Supplement 2 Secondary outcome measures**

The Fatigue Severity Scale (FSS) consists of 9 items rated on a scale from 1 (strongly disagree) to 7 (strongly agree) summarized in a mean score of all items (1). The PROMIS-Fatigue Short Form 8a consists of 8 items to be rated on a scale from 1 to 5, adding up to a total score between 8 and 40 (2). The PROMIS-SF 8a was found to have robust psychometric properties (3). Limitations in daily functioning were assessed by the Sickness Impact Profile (SIP8), consisting of 8 subscales measuring limitations on different domains of functioning (4, 5). Items are answered with yes or no, leading to a weighed score per subscale. The subscale scores are summed to a total score, with higher scores indicating more severe limitations (4, 5). The Work and Social Adjustment Scale (WSAS) was used to measure with 5 items the restrictions in 5 different domains of societal participation on a scale from 1 (not at all restricted) to 8 (severely restricted) (6). The total score (5-40) is the sum of all items. Health-related quality of life was assessed by the Short-Form 36 (SF36), consisting of 36 items and 8 subscales. Raw scores on each scale are converted to a score of 0 to 100, with a higher score indicating a higher level of well-being (7).

**References**

1. Krupp LB, LaRocca NG, Muir-Nash J, Steinberg AD. The fatigue severity scale. Application to patients with multiple sclerosis and systemic lupus erythematosus. Arch Neurol. 1989;46(10):1121-3.

2. Cook KF, Bamer AM, Roddey TS, Kraft GH, Kim J, Amtmann D. A PROMIS fatigue short form for use by individuals who have multiple sclerosis. Quality of life research : an international journal of quality of life aspects of treatment, care and rehabilitation. 2012;21(6):1021-30. doi:10.1007/s11136-011-0011-8

3. Kamudoni P, Johns J, Cook KF, et al. Standardizing fatigue measurement in multiple sclerosis: the validity, responsiveness and score interpretation of the PROMIS SF v1.0 - Fatigue (MS) 8a. Multiple sclerosis and related disorders. 2021;54:103117. doi:10.1016/j.msard.2021.103117

4. Jacobs HM, Luttik A, Touw-Otten FW, de Melker RA. [The sickness impact profile; results of an evaluation study of the Dutch version]. Ned Tijdschr Geneeskd. 1990;134(40):1950-4.

5. Bergner M, Bobbitt RA, Carter WB, Gilson BS. The Sickness Impact Profile: development and final revision of a health status measure. Med Care. 1981;19(8):787-805.

6. Thandi G, Fear NT, Chalder T. A comparison of the Work and Social Adjustment Scale (WSAS) across different patient populations using Rasch analysis and exploratory factor analysis. J Psychosom Res. 2017;92:45-8. doi:10.1016/j.jpsychores.2016.11.009

7. Aaronson NK, Muller M, Cohen PD, et al. Translation, validation, and norming of the Dutch language version of the SF-36 Health Survey in community and chronic disease populations. J Clin Epidemiol. 1998;51(11):1055-68.
